# Cardiac Damage in Hypertension: From Molecular Mechanisms to Novel Therapeutic Approaches

**DOI:** 10.3390/ijms26125610

**Published:** 2025-06-11

**Authors:** Giovanna Gallo, Speranza Rubattu

**Affiliations:** 1 Department of Clinical and Molecular Medicine, Sapienza University of Rome, Via di Grottarossa 1035–1039, 00189 Rome, Italy; giovanna.gallo@uniroma1.it; 2 Cardiology Unit, Sant’Andrea University Hospital, Via di Grottarossa 1035–1039, 00189 Rome, Italy; 3 IRCCS Neuromed, 86077 Pozzilli, Italy

**Keywords:** hypertension-mediated organ damage, cardiac hypertrophy, oxidative stress, mitochondrial damage, inflammation

## Abstract

Cardiac hypertrophy represents a central manifestation of hypertension-mediated organ damage (HMOD), which consists of structural and functional changes as a response to sustained pressure overload. Oxidative stress and inflammation play central roles in the development of cardiac hypertrophy, contributing to myocardial remodeling in association with mechanical stress and neurohormonal activation. The imbalance between the production of reactive oxygen species and antioxidant defense mechanisms is associated with the activation of signaling pathways and the expression of genes involved in the development and progression of cardiac fibrosis and hypertrophy. Oxidative stress is also related to mitochondrial dysfunction, redox-sensitive transcription factors, post-translational modifications, and epigenetic modulation. Novel therapeutic strategies can target these molecular pathways, reducing the impact of hypertension on HMOD. Type-2 sodium glucose transporter inhibitors were shown to restore mitochondrial bioenergetics, reducing oxidative stress, and suppressing inflammation. Also, glucagon-like peptide-1 receptor agonists reduce ROS generation and stabilize mitochondrial structure and function. In addition, vericiguat, which represents an approach targeted to restore nitric oxide-soluble guanylate cyclase signaling, might represent a valuable therapeutic approach, working to prevent and slow the progression of cardiac hypertrophy before the development of heart failure. In this review we will describe the pathophysiological mechanisms associated with cardiac hypertrophy and discuss the recent innovative therapeutic strategies with potential implications for prevention and management.

## 1. Introduction

Hypertension represents one of the most important cardiovascular risk factors, being associated with a significant increase in the risk of major cardiovascular events (MACEs), such as myocardial infarction (MI), stroke, heart failure (HF), death from cardiovascular causes, and major kidney disease events, including a progressive reduction in estimated glomerular filtration rate (eGFR), the initiation of long-term kidney-replacement therapy, and death from kidney-related causes [1]. In this context, the development of hypertension-mediated organ damage (HMOD), which consists of structural and functional changes in organs adversely affected by hypertension, including the heart, brain, kidneys, eyes and vessels, has been associated with further increases in the risks of MACEs and cardiovascular mortality [2].

Cardiac hypertrophy is a central manifestation of HMOD, representing both an adaptive and maladaptive response to sustained pressure overload [3]. While initially compensatory, prolonged hypertrophy leads to myocardial fibrosis, diastolic dysfunction, and ultimately HF. Oxidative stress plays a central role in this progression by modulating signaling pathways, mitochondrial function, protein homeostasis, and gene expression [4].

Reactive oxygen species (ROS) are normally produced as byproducts of cellular metabolism and serve essential signaling roles [5]. However, under pathological conditions such as hypertension, an imbalance occurs between ROS production and antioxidant defense mechanisms. This shift results in oxidative stress, a key driver of endothelial dysfunction, vascular inflammation, and cardiac fibrosis. Moreover, oxidative stress acts synergistically with mechanical stress and neurohormonal activation to exacerbate cardiac hypertrophy and myocardial remodeling [6].

An increasing body of evidence has elucidated several molecular mechanisms by which oxidative stress mediates HMOD, including mitochondrial dysfunction, activation of NADPH oxidase isoforms, redox-sensitive transcription factors, oxidative post-translational modifications (Ox-PTMs), and epigenetic modulation [7]. This knowledge has expanded the understanding of cardiac pathology in hypertension beyond pressure overload, highlighting the central role of oxidative stress and inflammation in disease progression [8].

In this review, we will focus on the pathophysiological mechanisms associated with the oxidative stress and inflammation underlying left ventricular hypertrophy (LVH), the principal manifestation of cardiac HMOD, and on recent innovative therapeutic strategies able to target these molecular pathways and to offer powerful tools to combat hypertensive cardiac damage and its dramatic consequences.

## 2. Signaling Pathways Underlying ROS-Mediated Development of HMOD

A dysregulated production of ROS, including superoxide anion (O_2_^•−^), hydrogen peroxide (H_2_O_2_), and hydroxyl radicals (•OH), leading to the disruption of cellular homeostasis, has been associated with detrimental structural and functional alterations in the cardiovascular system [9,10].

In the context of hypertension, ROS amplify intracellular signaling through the oxidation of redox-sensitive cysteine residues in kinases and phosphatases. One prominent class of affected proteins includes mitogen-activated protein kinases (MAPKs), such as extracellular signal-regulated kinases (ERK1/2), p38 MAPK, and c-Jun N-terminal kinase (JNK) [11]. These kinases orchestrate key processes, including cellular proliferation, hypertrophy, inflammation, and the apoptosis involved in cardiac remodeling. Oxidative modification of upstream regulators, such as ASK1 and c-Src, potentiates MAPK activation, resulting in a vicious circle that sustains redox signaling and hypertrophy [12].

The phosphatidylinositol 3-kinase (PI3K)/Akt axis is also modulated by ROS, which enhance its activation through the inhibition of phosphatase and tensin homolog (PTEN) and the activation of receptor tyrosine kinases like VEGFR and PDGFR, therefore contributing to the development and progression of LVH [13].

In addition, ROS influence ion channels’ functions, particularly in the context of calcium signaling [14]. Indeed, voltage-gated calcium channels, IP3 receptors, and store-operated calcium entry mechanisms represent targets of oxidative modulation. ROS-sensitive transient receptor potential (TRP) channels, in particular TRPM2 and TRPM7, are activated under oxidative stress and facilitate abnormal Ca^2+^ influx [15]. This altered calcium handling contributes not only to contractile dysfunction but also to the transcriptional activation of hypertrophy genes.

Moreover, the oxidative inhibition of sarcoplasmic reticulum calcium ATPases and ryanodine receptors (RyR_2_) disrupts excitation–contraction coupling, promoting arrhythmogenic potential and myocardial injury [16]. These redox-sensitive alterations in calcium homeostasis represent an important mechanistic bridge between oxidative stress and the functional deterioration observed in hypertensive hearts.

The interplay between oxidative and calcium signaling creates a complex network that modulates cardiomyocyte phenotype and function. Redox-sensitive calcium/calmodulin-dependent protein kinase II (CaMKII) is a major actor of these pathways [17]. ROS-induced oxidation of methionine residues in CaMKII maintains its constitutive activation, which in turn phosphorylates downstream targets involved in calcium cycling, transcriptional activation, and apoptosis. CaMKII-mediated phosphorylation of phospholamban (PLN) enhances sarcoplasmic reticulum Ca^2+^ uptake, whereas hyperactivation can lead to calcium overload and mitochondrial stress [18]. Moreover, CaMKII amplifies inflammatory signaling through NF-κB and NLRP3 inflammasome activation, reinforcing pathological remodeling [19]. NF-κB translocates to the nucleus and promotes the expression of proinflammatory cytokines, adhesion molecules, and fibrotic mediators, thereby favoring cardiac remodeling [20].

This bidirectional amplification between redox stress and calcium imbalance constitutes a vicious circle that sustains and propagates HMOD, even in the absence of elevated blood pressure [21].

Other transcription factors, such as activator protein-1 (AP-1), signal transducer and activator of transcription 3 (STAT3), and the antioxidant response mediator nuclear factor erythroid 2–related factor 2 (Nrf2) are modulated by ROS through direct oxidation of cysteine residues or through upstream kinases [22]. In particular, Nrf2 plays a dual role as both a redox sensor and an antioxidant defense regulator [23]. Under basal conditions, Nrf2 is sequestered in the cytoplasm by KEAP1 and targeted for degradation. Oxidative modification of KEAP1 cysteines results in Nrf2 stabilization and nuclear translocation, where it binds to antioxidant response elements (AREs) to upregulate gene expression of HO-1, SOD, and catalase. While this pathway is protective, some evidence suggests that Nrf2 activation is insufficient under sustained hypertensive stress, thus representing a potential therapeutic target [24].

Experimental studies have shown the fundamental role of bromodomain-containing protein 4 (BRD4), a transcriptional coactivator that responds to ROS by promoting chromatin remodeling and the expression of TGF-β1/SMAD pathway components [25,26]. BRD4 inhibition reduced cardiomyocyte hypertrophy and oxidative stress through the attenuation of NF-κB and the enhancement of Nrf2/HO-1 signaling [27].

Apart from altering gene expression, ROS exert also direct effects on protein function through oxidative post-translational modifications (Ox-PTMs) [28]. Reversible oxidation of cysteine thiol groups can result in sulfenylation, S-glutathionylation, and S-nitrosylation, whereas irreversible modifications such as sulfonylation lead to protein inactivation and degradation. These modifications affect a range of proteins involved in cytoskeletal organization, contractile function, and metabolic regulation [29]. In hypertension, within the myocardium, proteins like ATP synthase, RyR2, and thioredoxin are subject to Ox-PTMs that compromise mitochondrial ATP production and redox buffering capacity [30]. S-nitrosylation of endothelial nitric oxide synthase (eNOS) in critical cysteine residues disrupts NO production and contributes to vascular dysfunction [30]. Additionally, oxidative nitration of tyrosine residues, forming 3-nitrotyrosine, was observed in hypertensive models, although its functional consequences remain to be fully elucidated.

The oxidative inactivation of phosphatases like PP2Acα leads to persistent activation of hypertrophic pathways [31]. In this context, proteasomal regulators such as REGγ promote degradation of PP2Acα, driving the nuclear export of FOXO3a, downregulation of MnSOD, and further ROS amplification [32]. Restoration of MnSOD using mimetics such as MnTBAP was shown to reverse this phenotype, confirming a critical role for ROS-regulated proteostasis in cardiac hypertrophy [33].

**Figure 1:**
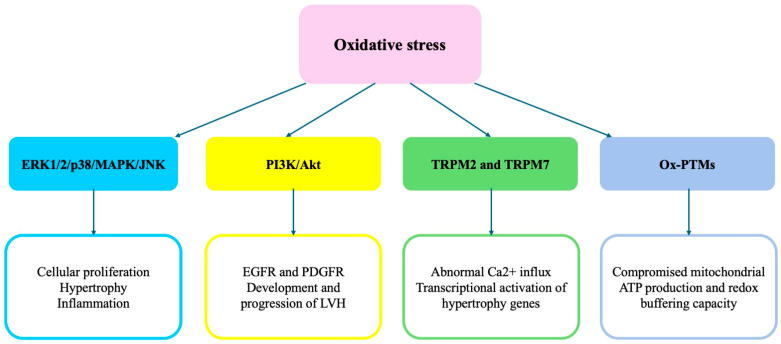
Summary of the most relevant signaling pathways involved in the ROS-mediated development of cardiac hypertrophy.

Figure 1 summarizes the signaling pathways involved in the ROS-mediated development of cardiac hypertrophy. (See the text for explanations of abbreviations).

## 3. Role of Mitochondrial Dysfunction

Mitochondria are both producers and the targets of ROS. In hypertension, mitochondrial oxidative phosphorylation is compromised, particularly at the level of complex I (NADH dehydrogenase). Deficiency or dysfunction of complex I subunits results in impaired electron transport, leading to excess leakage of electrons and ROS generation. Mitochondrial ROS (mtROS) initiate a feed-forward loop that exacerbates mitochondrial damage, disrupts membrane potential, and impairs ATP production [34]. Previous evidence showed that reduced expression of either Ndusf1 or Ndufs4 complex I subunits led to cardiac hypertrophy [35,36].

We demonstrated that Ndufc2, another complex I subunit, plays a key role in cardiac hypertrophy. In fact, Ndufc2-silenced cardiomyocytes developed a significant degree of hypertrophy that was associated with an increased expression level of hypertrophy markers (ANP and β-MHC) [37]. Exposure to nicotinamide, with the aim of rescuing complex I function, reduced cellular volume and the gene expression levels of both ANP and β-MHC. The expression of MnSOD and phospho-AMPK was reduced, with a parallel increase of phospho-AKT, in Ndufc2-silenced H9c2 cells. In this context, a reduced expression of SIRT3 was also observed [37]. SIRT3 was previously demonstrated to counteract cardiac hypertrophy in primary cultures of cardiomyocytes by activating MnSOD and catalase, thereby decreasing cellular levels of ROS, suppressing Ras activation and the downstream signaling pathway through the MAPK/ERK and PI3K/Akt pathways. SIRT3 also repressed the activities of transcription factors like GATA4 and NFAT, and of translation factors such as eukaryotic initiation factor 4E (elf4E) and the ribosomal protein S6 kinase (PS6K) [38,39].

We have also reported that two variants in the human gene were significantly associated with the presence of LVH in a cohort of hypertensive patients [37]. In particular, the carrier status of either the A allele at NDUFC2/rs641836 or the TT genotype at NDUFC2/rs11237379, both associated with reduced Ndufc2 expression, was associated with significantly increased cardiac wall thickness and LV mass, independently of blood pressure levels, as well as anthropometric, clinical, and pharmacological parameters. Moreover, the combined presence of the A allele at rs641836 and TT genotype at rs11237379 was associated with increased LVH.

Consistently with the in vitro evidence, patients carrying the TT genotype at rs23117379 were characterized not only by a reduced NDUFC2 expression, but also by a marked downregulation of SIRT3 expression [37].

These data reinforced the evidence that complex I-dependent mitochondrial dysfunction plays a contributory role in cardiomyocytes hypertrophy and have provided the first evidence that this molecular mechanism contributes to LVH development in human hypertension [37].

Cardiomyocyte adaptation to hypertrophic stress requires not only sufficient ATP supply but also robust mitochondrial quality control [40]. An efficient mitochondrial dynamic (biogenesis, fusion/fission and mitophagy) is essential to sustain energetic demands and prevent the accumulation of damaged organelles [40]. Mitophagy, the selective degradation of damaged mitochondria via autophagy, is essential to prevent the accumulation of dysfunctional ROS-producing organelles. A dysregulated mitophagy has been observed in hypertensive hearts with either insufficient or excessive activation, depending on disease context and stage [41].

Defective mitophagy impairs mitochondrial turnover, leading to accumulation of damaged mtDNA and increased ROS [42]. Excessive mitophagy, on the other hand, depletes healthy mitochondria, compromising ATP generation. Thus, a delicate balance in mitophagy-related activity is essential for mitochondrial integrity in hypertension [43].

The PINK1-Parkin pathway is the most well-characterized mitophagy axis. Under mitochondrial depolarization, PINK1 accumulates on the outer mitochondrial membrane and recruits Parkin, which ubiquitinates mitochondrial proteins to signal for autophagic clearance [44]. In FoxP3 knockout mice, pathological upregulation of Parkin-mediated mitophagy contributes to cardiac remodeling [45]. Conversely, overexpression of mitochondrial S-nitrosoglutathione reductase (mtGSNOR) attenuates excessive mitophagy and protects against hypertrophy [46].

Different studies have uncovered the role of proteins such as MTG1 and TIM50 in mitochondrial translation and protein import. MTG1, needed for mitochondrial ribosome assembly, is protective against pressure-overload-induced cardiac hypertrophy. Conversely, its deficiency exacerbates mitochondrial dysfunction, ROS production, and activation of pro-hypertrophic MAPK pathways [47]. TIM50, a component of the translocase of the inner membrane (TIM) complex, facilitates protein import into the mitochondrial matrix. Downregulation of TIM50 in hypertrophied hearts correlates with increased oxidative stress, ASK1/JNK/p38 MAPK activation, and mitochondrial dysfunction [48].

The endoplasmic reticulum (ER) is a central organelle in the maintenance of protein folding, lipid biosynthesis, and calcium storage [49]. The ER acts as a dynamic signaling hub, critically linked to mitochondrial function and cellular homeostasis. In the setting of hypertension, persistent mechanical and oxidative stress disrupts ER function, leading to ER stress and activation of the unfolded protein response (UPR). This disruption contributes to maladaptive remodeling of the myocardium by initiating apoptotic pathways, altering calcium homeostasis, and increasing ROS generation [50].

Under physiological conditions, the ER chaperones facilitate correct protein folding and quality control. However, when the protein load exceeds folding capacity—such as during oxidative or hypertrophic stress—the ER initiates a compensatory mechanism termed the UPR. This response is coordinated by three principal sensors: inositol-requiring enzyme 1 (IRE1), protein kinase R-like ER kinase (PERK), and activating transcription factor 6 (ATF6) [51].

Activation of the UPR initially aims to restore ER function by upregulating chaperone proteins, inhibiting global protein translation, and enhancing degradation of misfolded proteins [52]. However, chronic or excessive ER stress leads to the apoptosis, inflammation, and tissue remodeling that are hallmarks of cardiac pathology in hypertension [51,52].

Oxidative stress exacerbates ER dysfunction through disulfide bond mis-formation and thiol oxidation of ER-resident proteins [53]. Notably, the ER oxidoreductin 1 (Ero1)-PDI complex not only regulates redox-dependent folding but also generates H_2_O_2_ as a byproduct, creating a self-sustaining source of oxidative stress within the ER [54].

The ER communicates closely with mitochondria through specialized microdomains termed mitochondria-associated membranes (MAMs) [55]. These structures coordinate calcium and lipid exchange and serve as platforms for redox signaling. In hypertension, the MAM interface becomes pathologically activated, promoting excessive calcium transfer from the ER to mitochondria. This results in mitochondrial calcium overload, loss of membrane potential, and increased ROS production [56].

In HF models, natriuretic peptides stimulate autophagy/mitophagy in cardiomyocytes and reduce structural and functional mitochondrial damage, suggesting a promising role for mitochondria as a therapeutic target, including in hypertensive patients, to prevent HF [57,58].

**Figure 2:**
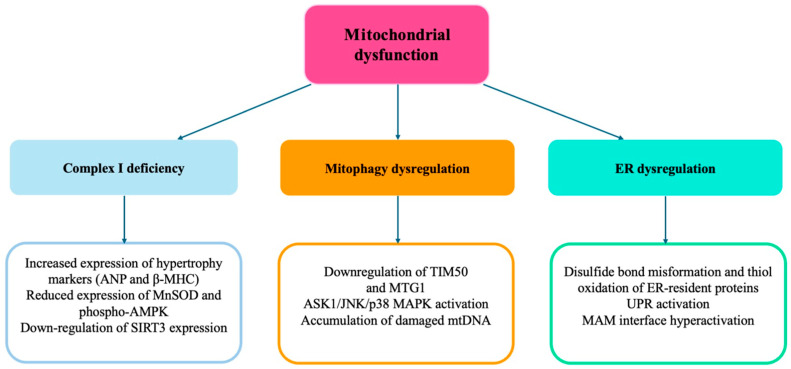
Summary of the mechanisms dependent on mitochondrial dysfunction involved in cardiac HMOD.

Figure 2 summarizes the role of mitochondrial dysfunction in the development of cardiac HMOD. (See the text for explanations of the abbreviations).

## 4. Epigenetic Modulation

Oxidative stress acts as a potent epigenetic modulator, altering the transcriptional landscape in cardiomyocytes and vascular cells through reversible and irreversible changes in DNA structure, histone modifications, and non-coding RNA expression [59]. These epigenetic shifts contribute to sustained activation of hypertrophic, pro-fibrotic, and inflammatory gene programs even after the initial hypertensive insult has subsided [59].

Histone proteins are subject to a variety of post-translational modifications (PTMs), including acetylation, methylation, phosphorylation, and ubiquitination. These modifications influence chromatin accessibility and thereby control gene transcription [60]. Oxidative stress modifies the activity of key histone-modifying enzymes such as histone acetyltransferases (HATs), which promote transcriptional activation by relaxing chromatin, and histone deacetylases (HDACs), which mediate transcriptional inhibition [61]. Moreover, ROS-mediated signaling can modulate the assembly and recruitment of chromatin remodelers to target loci, reinforcing gene expression changes linked to fibrosis, apoptosis, and mitochondrial biogenesis. For instance, oxidative stress promotes chromatin accessibility at the NF-κB-promoter regions, enhancing inflammatory transcriptional programs in cardiac tissue [62].

ROS also directly affect DNA integrity and methylation. These modifications alter the expression of key regulatory genes in oxidative metabolism and hypertrophy [63]. The hypermethylation of promoters for antioxidant enzymes like SOD2 and CAT under oxidative stress have been documented in hypertensive models, suggesting a feed-forward loop that exacerbates redox imbalance [64].

The reduction in the DNA methylation of transcription factors like GATA4 and myocyte enhancer factor 2C (Mef2C) can promote the expression of cardiomyocyte embryonic genes such as ANP, BNP, endothelin-1 and β-MHC, and can favor cardiomyocyte hypertrophy. Moreover, the overexpression of the HAT CREB-binding protein/p300 and the inhibition of HDACs silencing the fetal gene program in the adult heart were described in models of cardiac hypertrophy [65].

Epigenetic and redox-sensitive mechanisms also regulate microRNAs (miRNAs) involved in cardiac HMOD, such as miR-200c, which affects sarcomere organization and promotes cardiomyocytes hypertrophy; miR-210, which inhibits mitochondrial respiration and enhances ROS generation; miR-106a and miR-28, the dysregulation of which alters mitochondrial fusion and function, leading to mitochondrial depolarization and increased ROS [66].

Long non-coding RNAs (lncRNAs) and circular RNAs (circRNAs) were also shown to interact with histone modifiers and transcription factors, modulating cardiac gene expression in response to stress [67]. ROS can influence lncRNA expression by activating redox-sensitive transcription factors (e.g., NF-κB, HIF-1α), which bind to lncRNA promoters. These lncRNAs may bind to chromatin-modifying complexes or sequester miRNAs to regulate gene expression [68].

**Figure 3:**
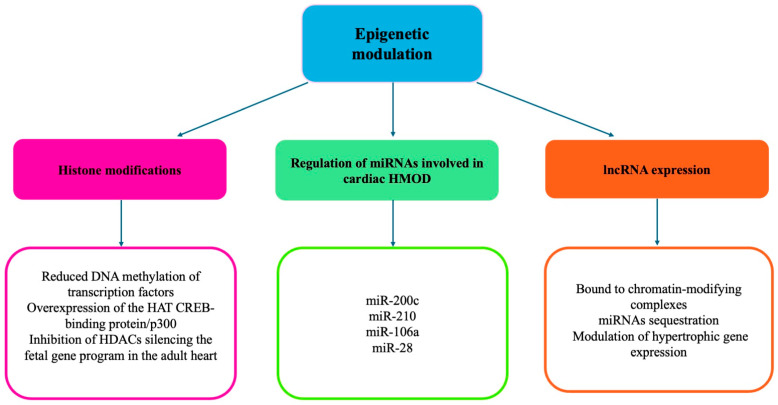
Summary of the mechanisms dependent on the epigenetic modulation of cardiac hypertrophy development.

Figure 3 summarizes the role of epigenetic modulation in the development of cardiac hypertrophy. (See the text for explanations of abbreviations).

## 5. Role of Inflammation

Inflammation is a key driver of HMOD, and it intersects with redox pathways through inflammasome activation [69]. The NLRP3 inflammasome integrates signals from oxidative stress and cellular damage to initiate caspase-1-mediated cleavage of IL-1β and IL-18. This process promotes fibrosis, apoptosis, and ventricular dysfunction [70].

ROS serve as second messengers that trigger NLRP3 activation via different mechanisms, including thioredoxin depletion, TRP channel activation, and NOX-derived signaling [71]. Therapeutic inhibition of NLRP3, using agents like MCC950 or upstream suppressors such as SGK1 inhibitors (e.g., EMD638683), has demonstrated efficacy in reducing angiotensin II-induced hypertrophy and fibrosis [72].

MCC950 also reduced the levels of IL-1β, IL-18, vascular cell adhesion molecule 1 (VCAM-1), and intercellular adhesion molecule 1 (ICAM-1), alleviating myocardial fibrosis and infiltration of inflammatory cells [73].

Moreover, toll-like receptor 4 (TLR4) activation by ROS in cardiomyocytes enhances NF-κB signaling and exacerbates mitochondrial dysfunction [74]. Inhibition of TLR4 using antagonists like lipopolysaccharide (RS-LPS) has been shown to prevent cardiac hypertrophy and preserve mitochondrial function in preclinical models, reinforcing the importance of inflammatory–redox crosstalk [75,76].

In animal models, chronic administration of colchicine was shown to attenuate cardiac hypertrophy [77,78].

Proinflammatory pathways are also activated in conditions which often coexist with hypertension, such as obesity and diabetes [79,80]. Excessive lipid accumulation contributes to cardiac lipotoxicity, mitochondrial dysfunction, and oxidative stress. Proteins like PLIN5 and CTRP9, which regulate lipid metabolism and oxidative capacity, are downregulated in hypertrophic hearts, leading to reduced fatty acid oxidation and enhanced ROS generation [81,82]. Lipid-induced hypertrophy is also associated with suppression of AMPK and activation of mTOR, two key regulators of metabolic signaling and mitochondrial health. Restoration of AMPK activity via CTRP9 alleviates oxidative stress and improves cardiac energetics, underscoring the pivotal role of metabolic–redox integration in hypertrophy [83]. In diabetes, hyperglycemia intensifies oxidative stress through elevated glucose oxidation, activation of the polyol and hexosamine pathways, and suppression of mitochondrial antioxidants such as thioredoxin 2. This process culminates in increased calcium overload, ROS generation, and activation of pro-hypertrophic signaling [84].

The potential roles of pleiotropic stress-related differentiation factors, interleukins, TNF, and chemoattractant proteins as HMOD biomarkers is currently under investigation [85].

## 6. Therapeutic Perspectives

Among the emerging pharmacological classes, sodium-glucose co-transporter 2 inhibitors (SGLT2i), glucagon-like peptide-1 receptor agonists (GLP-1RA), and soluble guanylate cyclase (sGC) stimulators such as vericiguat might have a potential efficacy in reducing the progression and reversing the features of organ damage, due to their molecular actions. While originally developed for metabolic or vascular indications, these agents have shown a remarkable capacity to ameliorate redox imbalance, restore mitochondrial health, and disrupt the maladaptive cellular signaling implicated in hypertensive heart disease [86].

SGLT2i were shown to modulate cardiac oxidative stress and mitochondrial dysfunction [87]. SGLT2i restore mitochondrial membrane potential and improve the function of electron transport chain complexes, particularly complexes I and III, thereby enhancing ATP production [88]. This improvement in bioenergetic efficiency is associated with enhanced mitochondrial calcium handling, an effect partly mediated through the inhibition of the sodium–hydrogen exchanger 1 (NHE1) [89]. By reducing intracellular sodium, SGLT2i prevent secondary calcium overload, preserving mitochondrial integrity and reducing oxidative burden. In addition, SGLT2i decrease the expression of serum- and glucocorticoid-responsive kinase-1 (SGK1), which modulates the expression of many ion channels contributing to fibrosis, ventricular remodeling and hypertrophy [90]. SGLT2i also attenuate the activity of NADPH oxidase isoforms NOX2 and NOX4, reducing the generation of superoxide and hydrogen peroxide. In parallel, SGLT2i increase NAD+ availability and stimulate AMPK phosphorylation, promoting fatty acid oxidation and further stabilizing mitochondrial dynamics [91].

Different studies have demonstrated consistent reductions in both LV mass and wall thickness, even in non-diabetic individuals, under SGLT2i treatment. The EMPA-HEART Cardiolink-6 trial showed a significant reduction in LV mass, assessed with cardiac magnetic resonance, after 6 months of treatment with empagliflozin in diabetic patients with coronary artery disease [92]. Consistent results were obtained in the DAPA-LVH study in patients treated with dapagliflozin [93]. This effect was mediated by downregulation of pro-hypertrophic pathways such as Akt/mTOR and MAPK (including ERK, p38, and JNK) [94]. In addition, SGLT2i modulate TGFβ1/Smad signaling in a glycemic-independent manner and attenuate angiotensin II-induced myocardial fibrosis and collagen synthesis [95]. The doses of both empagliflozin and dapagliflozin used in human studies were 10 mg/kg/day, once daily. The dosages ranged between 10 and 20 mg/kg/day in the preclinical studies.

Different studies have shown that the pleiotropic effects of SGLT2i are mediated both by a state of relative glucose deprivation, due to glucosuria, and by a direct effect on mitochondrial homeostasis. A shift in energetic substrate occurs, with an increase in ketone production and a decreased amount of electron donors such as NADH and FADH2, resulting in a reduction in mitochondrial ROS. On the other hand, SGLT2i restored mitochondrial morphology and biogenesis, normalizing the levels of proteins regulating fission and fusion, such as optic atrophy 1 (OPA1) and mitofusin 2 (Mfn2) [96,97].

Similarly, GLP-1 receptor agonists demonstrated protective effects against oxidative stress and organ damage in hypertension. GLP1-R agonists exerted anti-inflammatory effects through the activation of peroxisome proliferator-activated receptor γ (PPARγ), resulting in the inhibition of intracellular serine/threonine kinases (like JNK) and in a reduction in insulin receptor substrate (IRS)-1 serine phosphorylation [98]. GLP-1R activation upregulates genes involved in mitochondrial biogenesis and improves oxidative phosphorylation by increasing the expression of peroxisome proliferator-activated receptor gamma coactivator 1-alpha (PGC-1α) and nuclear respiratory factors. This is accompanied by suppression of NOX4 expression, resulting in a reduction in mitochondrial ROS production and preservation of the mitochondrial cristae morphology [99]. Cardiomyocyte hypertrophy is also mitigated by GLP-1RA through the modulation of histone deacetylase 4 (HDAC4), resulting in reduced expression of ANP and β-MHC. Furthermore, GLP-1RA enhance endothelial nitric oxide production via PI3K/Akt-mediated phosphorylation of eNOS, restoring vasodilation and limiting vascular remodeling [100]. Direct liraglutide treatment in isolated aged rat cardiomyocytes was able to restore the depolarized mitochondrial membrane potential, reduce ROS and nitrogen species, and normalize cytosolic Na^+^ levels [101]. Liraglutide treatment recovered the phospho-endothelial nitric oxide synthase (pNOS3) to NOS3 protein ratio in cardiomyocytes, leading to the inhibition of oxidative stress-induced injury via the IRS1-eNOS-PKG pathway [102].

GLP-1RA also downregulate TLR4 expression and NLRP3 inflammasome, thus suppressing downstream NF-κB activation and the production of cytokine and adhesion molecules [101]. This anti-inflammatory effect extends to the cardiac interstitium, where GLP-1RA blunt fibroblast activation and collagen deposition. Clinically, these effects might translate into improved diastolic function and reduced arterial stiffness [103].

The CMR substudy of the SUMMIT trial demonstrated that tirzepatide, a GIP/GLP-1 RA, led to reductions in LV mass and pericardiac adipose tissue, compared with placebo. The reductions in LV mass tended to correlate with changes in systolic blood pressure and were also associated with changes in LV end-diastolic volume, stroke volume, left atrium end-diastolic volume, and left atrium end-systolic volume [104].

Finally, vericiguat might represent a novel pharmacological approach by directly targeting impaired nitric oxide-soluble guanylate cyclase (NO-sGC) signaling, which is disrupted in oxidative-stress conditions [105]. In settings involving hypertension and HF, oxidative degradation of NO and oxidative modification of sGC reduce cGMP production, impairing vasodilation, mitochondrial biogenesis, and cardiomyocyte relaxation. Vericiguat acts by stimulating sGC independently of NO, thereby restoring cGMP levels even under oxidative conditions [106]. Elevated cGMP activates protein kinase G (PKG) which phosphorylates titin and improves myocardial compliance, counteracting the stiffening related to hypertrophic cardiac remodeling [107]. Additionally, cGMP–PKG signaling inhibits hypertrophic gene transcription, suppresses fibroblast proliferation, and enhances mitochondrial biogenesis by upregulating PGC-1α and SIRT1 [108]. Vericiguat also reduces myocardial oxidative stress indirectly by improving endothelial function and decreasing systemic vascular resistance, which relieves pressure overload [105].

Future studies are required to investigate the potential benefits of targeting NO-sGC signaling in the clinical setting of hypertension and HMOD. Further research should also evaluate the potential effects of the combinations of the abovementioned agents.

## 7. Conclusions

**Figure 4:**
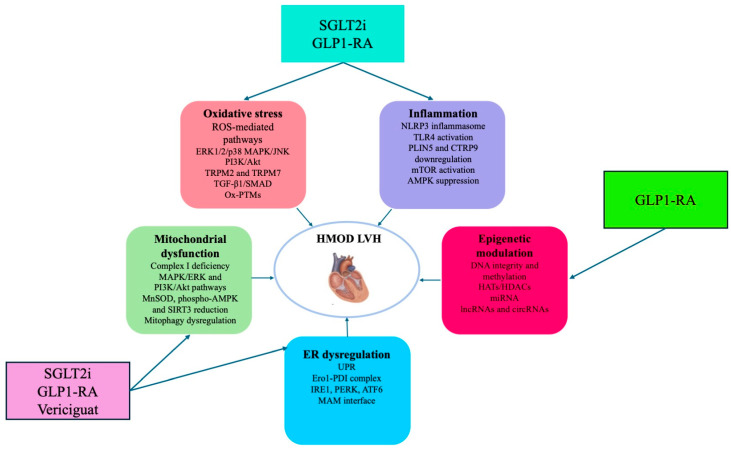
Schematic representation of the molecular mechanisms underlying the development of cardiac damage in hypertension that are discussed in this article. Novel therapeutic approaches [sodium-glucose co-transporter 2 inhibitors (SGLT2i), glucagon-like peptide-1 receptor agonists (GLP-1RA), and vericiguat as a soluble guanylate cyclase cGMP signaling stimulator] can target these molecular mechanisms, and these approaches allow for either the prevention or slowing of the progression of cardiac dysfunction before the development of HF. See the text for explanations of the abbreviations.

Oxidative stress, mitochondrial dysfunction and inflammation emerge as key mechanisms in the development and progression of HMOD (Figure 4).

Different novel therapeutic strategies can target these molecular pathways, reducing the impact of hypertension on cardiac damage. SGLT2i have shown remarkable efficacy in restoring mitochondrial bioenergetics, reducing oxidative stress, and reversing cardiac remodeling through mechanisms involving the improvement of mitochondrial functions and the suppression of inflammasome. GLP-1 receptor agonists exert synergistic effects, reducing ROS generation and stabilizing mitochondrial structure and function. In addition, vericiguat, which represents a targeted approach aiming to restore NO–sGC–cGMP signaling, might represent a future therapeutic approach used to prevent and slow the progression of HMOD before the development of HF (Figure 4). These pharmacological approaches might pave the way for future innovations in precision cardiovascular medicine.
